# Formative assessments in medical education: a medical graduate’s perspective

**DOI:** 10.1007/s40037-013-0089-5

**Published:** 2013-10-16

**Authors:** Ahmed Abu-Zaid

**Affiliations:** College of Medicine, Alfaisal University, PO Box 50927, Riyadh, 11533 Saudi Arabia

Dear Sir,

Generally speaking, there are two forms of assessment in medical education: formative and summative. Formative assessments happen repeatedly throughout the academic semester. They offer valuable qualitative feedback on students’ current teaching–learning outcomes, and accordingly aid faculty to adjust prospective teaching methodologies in order to enhance students’ subsequent learning outcomes **[** 1 **]**. Broadly, formative assessments are administered to: (1) pinpoint students’ areas of strengths and weaknesses, (2) steer prospective directions in teaching and learning, and (3) support self-inspiration to acquire knowledge and skills away from assessment-driven motives.

Formative assessments may or may not be computably graded and contribute to the final course grade. They are ‘good’ when minimally graded and ‘best’ when not computably graded at all. When they are minimally graded or not graded at all, students will have the chance to promote evolution of the learning process from merely scoring grades into a process of vivid, productive and dynamic educational experience. By doing so, students, in turn, will have an opportunity to: (1) explore a subject in a more thoughtful fashion, (2) be more investigational in their learning endeavours, (3) invest efforts in creating a holistic understanding of subject, (4) have time to critically appraise preconceived concepts, (5) link new information to already pre-existing knowledge, (6) make associations across scientific disciplines, and (7) promote effective utilization of higher-order skills (critical thinking, problem solving, etc.) to generate comprehensive understanding of course content. In a sense, formative assessments without computable grades eventually support a deep learning attitude which is desired in learning medical sciences. Regardless of whether formative assessments are graded or not graded, students will still be enthusiastically involved in these assessments if they perceive them to be encouraging and supportive in advancing knowledge and skills qualitatively, as well as overall course grade quantitatively.

Formative assessments can be negatively perceived as being demanding. However, with the passage of time, this negative standpoint will be reversed and students will start recognizing the concept of formative assessment as a normal routine in academic life, an irreplaceable element of curriculum and an unavoidable practice in medical education **[** 2 **]**. Moreover, formative assessments will eventually have a fruitful influence on students by offering ongoing feedback on their teaching–learning outcomes and suggestions as to how to progress further. Besides, integration of a rewarding bonus system into formative assessments is favourable and yields substantial benefits, such as: (1) encouraging a dynamic learning process, (2) relieving stress of summative assessments, and (3) most importantly enhancing the overall course grade.
